# Editorial: Zoonotic diseases: epidemiology, multi-omics, and host-pathogen interactions, volume II

**DOI:** 10.3389/fmicb.2025.1764511

**Published:** 2026-02-04

**Authors:** Thet Tun Aung, Yin Hong, Lei Deng

**Affiliations:** 1 Shanghai Veterinary Research Institute, Chinese Academy of Agricultural Sciences, Shanghai, China; 2 Ocular Anti-Infectives and Inflammation, Singapore Eye Research Institute, Singapore, Singapore; 3 Lanzhou Veterinary Research Institute, Chinese Academy of Agricultural Sciences, Lanzhou, China

**Keywords:** epidemiological surveillance, host–pathogen interactions, multi-omics analysis, one health, zoonotic pathogens

## Emerging and re-emerging zoonotic pathogens

Li, Qi et al. examined *Leucobacter holotrichiae* isolated from bovine abscesses and analyzed its genomic features, identifying virulence factors, antimicrobial resistance genes, and prophage elements. Their findings expand the known host range of this previously insect-associated bacterium and provide insights into its biological characteristics. Dehong et al. investigated *Streptococcus suis* across pig farms in eastern China and described infection prevalence, serotype distribution, virulence gene profiles, and seasonal trends. They also assessed pathogenic differences among serotypes using pig infection models. Sihag et al. conducted longitudinal molecular surveillance of *Orientia tsutsugamushi* in humans, rodents, shrews, and mites. They examined circulating serotypes, analyzed genetic diversity, and reported the first detection of a TA678-like strain in Puducherry, India. Zhang, Chen et al. analyzed the occurrence of *Cryptosporidium* spp., *Giardia duodenalis*, and *Enterocytozoon bieneusi* in crab-eating macaques. They characterized age-related infection patterns, identified zoonotic genotypes, and examined subtype diversity in *C. hominis*. Li, Li et al. investigated the lung microbiota of raccoon dogs using high-throughput sequencing and viral metagenomics. They characterized a diverse assemblage of bacterial, fungal, and viral taxa, including multiple zoonotic species, illustrating the complexity of pneumonia outbreaks in these animals.

## Mechanisms of pathogenesis and molecular evolution

Zhang, Yang et al. examined the role of Rab4b in the internalization of the *Glaesserella parasuis* cytolethal distending toxin (GpCDT). They analyzed Rab4b–CdtB interactions, investigated endosomal trafficking mechanisms, and identified EEA1 as a Rab4b-dependent regulator of GpCDT uptake. Wang, Guo et al. analyzed the distribution of the deubiquitinase gene elaD across 530 *Escherichia coli* strains. They characterized its prevalence in multiple serotypes, phylogenetic groups, and sequence types and investigated evolutionary relationships using phylogenomics. Yee et al. characterized *Streptococcus pluranimalium* isolates from aborted bovine fetuses. They analyzed genome sequences, examined phylogenetic relationships, and identified mobilome-associated genes and prophage integrations.

## Microbiome ecology in hosts and vectors

Akdur-Öztürk et al. investigated *Blastocystis* across humans, livestock, and environmental sources in rural Türkiye. They analyzed subtype distributions, examined host-specific patterns, and explored associations between *Blastocystis* colonization and gut microbial diversity. Wang, Yu et al. analyzed microbiome profiles in three tick species and investigated how infection with spotted fever group *Rickettsia* or *Anaplasma* influenced microbial community structure. They described pathogen-associated shifts in bacterial diversity and composition.

## Host responses and immunomodulation

Bi et al. examined host angiogenic responses triggered by *Echinococcus multilocularis*. They analyzed gene expression signatures, investigated the activation of the PDGFR/PI3K/AKT/FAK pathway, and described enhanced angiogenesis in infected liver tissues. Li, Liu et al. assessed the immunogenicity of extracellular products of *Pasteurella multocida* serotype A:3. They analyzed protein components, evaluated humoral and cellular immune responses, and examined protective efficacy against multiple serotypes in mice.

## Translational applications and vaccine development

Zhang, Chen et al. investigated the function of pipC in *Salmonella Enteritidis* and analyzed the phenotypic consequences of its deletion. They described reductions in environmental stress tolerance, downregulation of T3SS-associated genes, attenuation of virulence, and evaluated the ΔpipC strain as a live-attenuated vaccine candidate.

A broad conceptual foundation is outlined by Remya et al., who reviewed the epidemiology, transmission cycles, clinical manifestations, diagnostics, and public health relevance of zoonotic filariasis. Their work emphasized the interconnected nature of host, vector, and environmental factors that shape filarial disease dynamics.

Collectively, the studies in this Research Topic examined zoonotic pathogens across ecological, molecular, and immunological dimensions. By integrating epidemiology, genomics, transcriptomics, microbiome analysis, and mechanistic biology, these contributions advance the understanding of zoonotic disease emergence and host–pathogen interactions. The insights gained support the development of improved surveillance strategies, mechanistic models, and intervention tools. We thank all authors and reviewers for their contributions and hope this Research Topic stimulates further interdisciplinary research to address the expanding challenges posed by zoonotic diseases.

